# Antihypertensive effect of giant embryo brown rice and pre‐germinated giant embryo brown rice on spontaneously hypertensive rats

**DOI:** 10.1002/fsn3.1137

**Published:** 2019-07-30

**Authors:** Xin Zhou, GuoChao Zhao, ShuangYan Sun, JianYue Li

**Affiliations:** ^1^ Development Center of Plant Germplasm Resources, College of Life Sciences Shanghai Normal University Shanghai China

**Keywords:** blood pressure, giant embryo brown rice, pre‐germinated giant embryo brown rice, renal function, spontaneously hypertensive rats

## Abstract

“Shangshida NO.5” is a giant embryo mutant resulting from giant embryo gene (*GE*) dysfunction in “Chao2‐10” rice. Here, we compared the antihypertensive effects of “Chao2‐10” brown rice (C2‐10), “Shangshida NO.5” brown rice (GER), and pre‐germinated “Shangshida NO.5” brown rice (PGER) in spontaneously hypertensive rats (SHR). Male SHR at 6 weeks of age were divided into four groups and were fed with (a) a control diet (control), (b) a 40% C2‐10‐supplemented diet (C2‐10), (c) a 40% GER‐supplemented diet (GER), or (d) a 40% PGER‐supplemented diet (PGER) for 8 weeks, and their physiological and biochemical parameters were measured. The results showed that the C2‐10‐, GER‐, and PGER‐supplemented diets significantly decreased systolic blood pressure (SBP) and diastolic blood pressure (DBP) during the experiment. At the end of the experimental period, the SBP and DBP of the C2‐10, GER, and PGER groups were 7.6, 23.3, and 31.1 mmHg and 9.8, 21.1, and 29 mmHg lower than those in the control group, respectively, suggesting the GER and PGER diets were better able to inhibit blood pressure elevation than the C2‐10 diet. The serum creatinine levels in the C2‐10, GER, and PGER groups and the blood urea nitrogen content in the PGER group were significantly lower than those of the control group, indicating that C2‐10‐, GER‐, and especially PGER‐supplemented diets improved renal function. In addition, the antioxidant activity of the C2‐10 group and especially of the GER and PGER groups also improved. The above results suggest that “Shangshida NO.5” rice, particularly pre‐germinated rice, is a good dietary supplement for preventing the development of hypertension.

## 1 INTRODUCTION

Hypertension is one of the most common lifestyle‐related diseases and has been found to be an important risk factor for cardiovascular disease and renal pathology. Hypertension can be caused by several factors, such as genetics, environment, diet, and lifestyle, and several types of medicines, including diuretics, β‐adrenoceptor blockers, angiotensin‐converting enzyme (ACE) inhibitors, calcium channel blockers, α‐adrenergic receptor blockers, vasodilators, and centrally acting drugs, can be used to treat hypertension (Perez & Musini, 2008). Nevertheless, the treatment of hypertension is a long‐term process, and it depends not only on drug therapy but also on a combination of lifestyle changes, such as limiting alcohol and reducing salt and fat intake. Recent studies have found that many foods and herbs contain natural ingredients with antihypertensive activities. Therefore, increasing efforts have been devoted to studying the effects of natural foods or their bioactive derivatives, such as garlic (Ried, Frank, & Stocks, 2010), tea (Negishi et al., 2004), peptides (Tokunaga et al., 2004), and vitamin C (McRae, 2006), as functional foods or bioactive compounds for the treatment and prevention of hypertension.

γ‐Aminobutyric acid (GABA), one of the major inhibitory neurotransmitters in the central nervous system (Curtis & Johnston, 1974), is found in peripheral tissues (Erdö, 1985), and it has several well‐known physiological functions, such as neurotransmission and the induction of hypotension, and it acts as a diuretic and tranquilizer (Akobs, Jaeken, & Gibson, 1993; Cohen, Navarro, Clemenceau, Baulac, & Miles, 2002; Mody, Dekoninck, Otis, & Soltesz, 1994; Wong, Bottiglieri, & Snead, 2003). GABA is commonly found in various foods, including vegetables and fruits. Recently, feeding with GABA‐rich foods such as green tea (Abe et al., 1995), fermented milk (Hayakawa et al., 2004), soy sauce (Yamakoshi et al., 2007), tomatoes (Yoshimura et al., 2009), pre‐germinated brown rice (Ebizuka, Ihara, & Arita, 2009), and GABA‐enriched brown rice (Kawakami, Yamada, Yamada, Nabika, & Nomura, 2018) has been reported to have an obvious inhibitory effect on the elevation of blood pressure in spontaneously hypertensive rats (SHR). Previous reports have also shown that feeding fermented milk containing GABA for 12 weeks significantly decreased blood pressure in patients with moderate hypertension (Inoue et al., 2003).

Rice is one of the most consumed cereal grains in the world, and it is cultivated in over 100 countries and serves as a staple food of more than half of the world's population. Brown rice, which is hulled directly from rough rice, consists of bran layers (6%–7%), an embryo (2%–3%), and an endosperm (approximately 90%) (Chen, Siebenmorgen, & Griffin, 1998). Compared to polished rice, brown rice not only is enriched in basic nutritional components but also contains more bioactive components, such as vitamin E, dietary fiber, and GABA (Saikusa, Horino, & Mori, 1994). A previous report demonstrated that rice bran, a byproduct of brown rice, had an antihypertensive effect in SHR (Ardiansyah et al., 2006). Giant embryo rice is a functional rice that has a larger embryo than conventional rice. Since 1981, more types of giant embryo rice have been reported (Nagasawa et al., 2013; Zhang et al., 2007). Previous reports showed that giant embryo brown rice contains more nutrients and bioactive compounds, such as GABA, vitamin E, essential amino acids, crude protein, crude fat, and trace elements than are found in normal brown rice (Seo et al., 2011; Zhang, Hu, Tang, Zhao, & Wu, 2005). A previous report showed that giant embryo brown rice has an obvious impact on the prevention or inhibition of hyperlipidemia (Lee, Kim, Kang, & Nam, 2007) and hyperglycemia (Chung, Kim, Rico, & Kang, 2014). However, to date, there have been no reports that systematically compare the antihypertensive effects of brown rice, giant embryo brown rice, and pre‐germinated giant embryo brown rice.

Previous studies showed that “Shangshida NO.5” giant embryo rice contains higher levels of GABA (Zhao, Xie, Wang, & Li, 2017) and vitamin E (Wang, Song, & Li, 2013) than the control rice, “Chao2‐10.” In this study, we compared the antihypertensive effects of diets supplemented with “Chao2‐10” brown rice, “Shangshida NO.5” giant embryo brown rice, and pre‐germinated “Shangshida NO.5” giant embryo brown rice in SHR. Moreover, we determined for the first time the effects of C2‐10, GER, and PGER on kidney function in SHR. The results from the above analyses strengthened our understanding of the antihypertensive effects of giant embryo rice.

## 2 MATERIALS AND METHODS

### 2.1 Animal experiments

Six‐week‐old male spontaneously hypertensive rats (SHR) were used in this study. The forty SHR were divided into four groups (*n* = 10): control diet (control), “Chao2‐10” brown rice diet (C2‐10), “Shangshida NO.5” giant embryo brown rice diet (GER), and pre‐germinated “Shangshida NO.5” giant embryo brown rice diet (PGER). The compositions of the experimental diets are shown in Table 1. The rats were housed under a 12‐hr light–dark cycle, and the room was maintained at 25–27°C with 40%–50% humidity. Rats were acclimated to the housing conditions for at least 1 week before the start of the experiments. The rats were given free access to fresh diet and drinking water. Body weight, blood pressure, and heart rate were measured at the day of the feed and once a week afterwards.

**Table 1:** Composition of the experimental diets (g/kg)

Ingredient (g/kg)	Control	C2−10	GER	PGER
Casein	140.000	140.000	140.000	140.000
Cornstarch	465.692	65.692	65.692	65.692
Corn dextrin	155.000	155.000	155.000	155.000
Sucrose	100.000	100.000	100.000	100.000
Cellulose	50.000	50.000	50.000	50.000
Soya oil	40.000	40.000	40.000	40.000
Vitamin mixture	10.000	10.000	10.000	10.000
Mineral mixture	35.000	35.000	35.000	35.000
Choline	2.500	2.500	2.500	2.500
L‐cystine	1.800	1.800	1.800	1.800
Tert‐butylhydroquinone	0.008	0.008	0.008	0.008
Different brown rice	0.000	400.000	400.000	400.000
Total	1,000.000	1,000.000	1,000.000	1,000.000

Note

The giant embryo rice germination process.

After harvest, rice seeds were naturally dried, processed into brown rice, and stored at 4°C. To obtain germinated brown rice, rice seeds were first dried in a 42°C oven for 24 hr, soaked in water at 28°C for 24 hr, and then kept at 28°C for 12 hr to germinate until a bulge developed in the germ or a budlet appeared.

Abbreviations: Control, control diet; C2‐10, “Chao2‐10” brown rice diet; GER, “Shangshida NO.5” giant embryo brown rice diet; PGER, pre‐germinated “Shangshida NO.5” giant embryo brown rice.

### 2.2 Blood pressure and heart rate measurements

The systolic blood pressure (SBP), diastolic blood pressure (DBP), and heart rate were measured in unanesthetized animals using the tail‐cuff method with a BP‐2006A automated noninvasive blood pressure meter (Softron Co.). Each of these parameters was measured three to five times for each animal, and the mean of at least three readings was regarded as the value of each parameter for the animal. To raise the body temperature of the animal, the animal was placed in a heated box (38°C) for approximately 10–15 min before measurement.

### 2.3 Sample collection

At the end of the treatment, the animals were anesthetized with chloral hydrate sodium (1 ml/100 g body weight), and blood samples were obtained from the abdominal aorta and transferred to centrifuge tubes with EDTA. All tubes were centrifuged at 7,700 *g* at 4°C for 10 min. The livers were promptly excised and washed with 1*PBS sodium. Both the plasma and kidney tissue samples were stored at −80°C until analysis.

### 2.4 Plasma and kidney parameters

The blood urea nitrogen (BUN), serum creatinine (SCR), norepinephrine (NE), rennin, aldosterone (ALD), angiotensin II (Ang II), superoxide dismutase (SOD), catalase (CAT), malondialdehyde (MDA), total antioxidant capacity (T‐AOC), total cholesterol (TC), and triacylglycerol (TG) levels were determined. Kidney SOD, CAT, MDA, and T‐AOC levels were also determined. BUN was detected by urease methods, SCR by picric acid methods, SOD by xanthine oxidase, MDA by thiobarbituric acid, and CAT and T‐AOC by colorimetric methods using commercially available kits (Nanjing Jiancheng). NE, rennin, ALD, and Ang II were measured with an ELISA immunoassay kit (Nanjing Jiancheng). All tests were performed according to the manufacturer's instructions.

### 2.5 Statistical analysis

Values are presented as the means ± standard errors. To evaluate the differences among the groups studied, one‐way ANOVA followed by the LSD test was used. Probability values of *p* < .05 and *p* < .01 were considered significant at 95% and 99% confidence intervals, respectively.

## 3 RESULTS

### 3.1 Effects of supplemented diets on body weight, blood pressure, and heart rate

**Figure 1:**
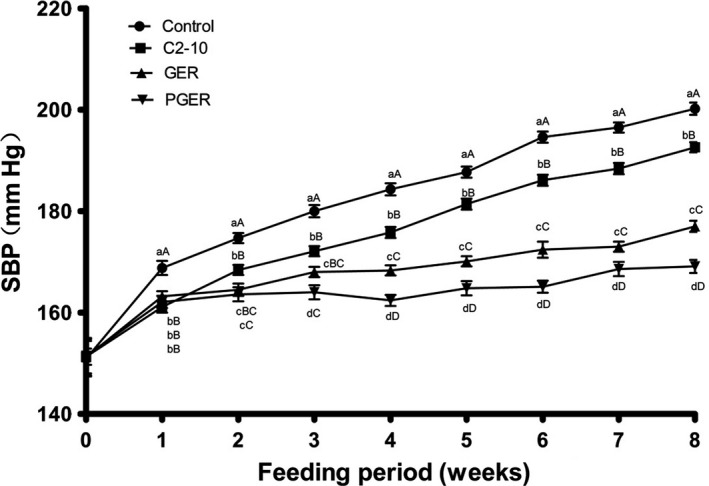
Effect of experimental diets on systolic blood pressure in rats. Control, control diet; C2‐10, “Chao2‐10” brown rice diet; GER, “Shangshida NO.5” giant embryo brown rice diet; PGER, pre‐germinated “Shangshida NO.5” giant embryo brown rice diet. ^a,b,c,d^ Mean values within a row with different superscript letters were significantly different (*p* < .05). ^A,B,C,D^ Mean values within a row with different superscript letters were significantly different (*p* < .01)

**Figure 2:**
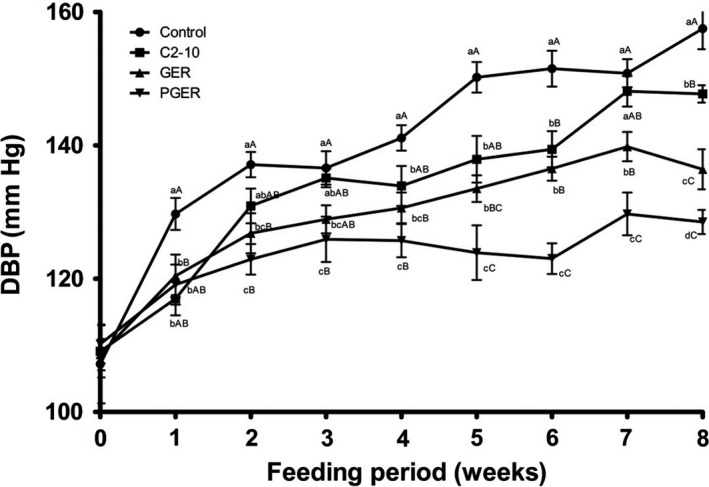
Effect of experimental diets on diastolic blood pressure in rats. Control, control diet; C2‐10, “Chao2‐10” brown rice diet; GER, “Shangshida NO.5” giant embryo brown rice diet; PGER, pre‐germinated “Shangshida NO.5” giant embryo brown rice diet. ^a,b,c,d^ Mean values within a row with different superscript letters were significantly different (*p* < .05). ^A,B,C,D^ Mean values within a row with different superscript letters were significantly different (*p* < .01)

In this study, the body weights of the four groups of SHR fed with different supplemented diets were similar during the experimental period (Table S1). However, a difference in the SBP levels among the four groups of SHR gradually developed during the experimental period (Figure 1). After 2 weeks, SHR fed with the control diet had the highest SBP among the four groups of SHR, followed by the SHR fed with C2‐10 (second highest SBP) and then GEB, while rats fed with PGER had the lowest SBP (Figure 1). This order in the SBPs level was constant until the end of the 8‐week experimental period. Similar results were also observed for DBP (Figure 2). At the end of the experiment, the SBP and DBP were 200.2 ± 1.2, 192.6 ± 1.1, 176.9 ± 1.2, and 169.1 ± 1.3 mmHg and 157.5 ± 2.8, 147.7 ± 1.4, 136.4 ± 2.4, and 128.5 ± 2.0 mmHg for the control, C2‐10, GER, and PGER diet groups, respectively. From the above experimental data, compared with the control diet, diets supplemented with either C2‐10, GER, or PGER significantly inhibited blood pressure elevation. We also found that the heart rate of the SHR was similar among the groups fed with different supplemented diets (Table S2).

### 3.2 Effects of supplemented diets on plasma levels of parameters related to the GABA hypotension pathway

To understand the effects of supplemented diets on plasma levels of parameters related to the GABA hypotension pathway, we assayed the levels of the related parameters, including NE, rennin, Ang II, and ALD (Table 2). We found that the NE and renin levels in the C2‐10, GER, and PGER groups were significantly lower than those in the control group (Table 2). For the plasma Ang II and ALD levels, compared with the control group, only the levels in the PGER group were considerably reduced (Table 2). In addition, compared with that of the C2‐10 group, plasma Ang II levels in the PGER groups were also significantly lower (Table 2). The results indicated that supplemented diets enriched with GABA, especially PGER, can improve plasma levels of parameters related to the GABA hypotension pathway.

**Table 2:** Levels of parameters related to the GABA hypotension pathway in plasma (*n* = 10, $\overset{¯}{x}\pm \text{SEM}$)

Parameters	Groups			
	Control	C2‐10	GER	PGER
NE (nmol/L)	14.20 ± 0.23aA	13.18 ± 0.42bAB	13.17 ± 0.37bAB	12.44 ± 0.22bB
Renin (ng/L)	172.21 ± 4.52A	156.37 ± 3.12B	149.96 ± 4.01B	144.04 ± 5.42B
Ang Ⅱ (ng/L)	277.15 ± 3.58aAB	280.75 ± 2.79aAB	267.76 ± 8.12abAB	258.78 ± 4.54bB
ALD (ng/L)	446.18 ± 12.42aA	412.5 ± 11.28abA	425.13 ± 12.01abA	410.05 ± 13.46bA

Abbreviations: Control, control diet; C2‐10, “Chao2‐10” brown rice diet; GER, “Shangshida NO.5” giant embryo brown rice diet; PGER, pre‐germinated “Shangshida NO.5” giant embryo brown rice diet.

^a,b,c^ Mean values within a row with different superscript letters were significantly different (*p* < .05).

^A,B,C^ Mean values within a row with different superscript letters were significantly different (*p* < .01).

### 3.3 Effects of supplemented diets on renal function and lipid levels

In this study, we also analyzed the effects of supplemented diets on renal function (BUN and SCR) and lipid levels (TC and TG). The SCR levels in the C2‐10, GER, and PGER groups were significantly lower than those in the control group (Table 3). The BUN contents in the PGER group were significantly lower than those in the control group, but the BUN levels among the control, C2‐10, and GER groups were similar (Table 3). The TG content in the PGER group was significantly lower than that in the control group (Table 3). The average TC levels in the control, C2‐10, GER, and PGER groups were in the order of control >C2‐10>GER>PGER, but there were no significant differences among them. The above data indicated that diets supplemented with brown rice, especially pre‐germinated giant embryo brown rice, can improve plasma renal function and lipid levels.

**Table 3:** Plasma renal function and lipid parameter levels (*n* = 10, $\overset{¯}{x}\pm \text{SEM}$)

Parameters	Groups			
	Control	C2‐10	GER	PGER
SCr (µmol/L)	61.79 ± 3.31aA	48.84 ± 2.95bAB	53.22 ± 2.22bAB	48.17 ± 1.28bB
BUN (mmol/L)	9.06 ± 0.23aA	8.60 ± 0.22aAB	8.81 ± 0.28aAB	7.84 ± 0.21bB
TG (mmol/L)	0.89 ± 0.03aA	0.89 ± 0.02aA	0.89 ± 0.02aA	0.78 ± 0.04bA
TC (mmol/L)	3.19 ± 0.41a	3.03 ± 0.33a	2.60 ± 0.26a	2.53 ± 0.21a

Abbreviations: Control, control diet; C2‐10, “Chao2‐10” brown rice diet; GER, “Shangshida NO.5” giant embryo brown rice diet; PGER, pre‐germinated “Shangshida NO.5” giant embryo brown rice diet.

^a,b,c^ Mean values within a row with different superscript letters were significantly different (*p* < .05).

^A,B,C^ Mean values within a row with different superscript letters were significantly different (*p* < .01).

### 3.4 Effects of supplemented diets on plasma and kidney antioxidant levels

To determine the effects of supplemented diets on plasma and kidney antioxidant levels, we measured the levels of plasma and kidney antioxidant parameters (SOD, CAT, MDA, and T‐AOC) in the present experiment. The detected plasma CAT levels in SHR in the control, C2‐10, GER, and PGER groups were similar (Table 4). However, the kidney CAT levels in the GER and PGER groups were significantly higher than those in the control SHR, and the kidney CAT levels in the control and C2‐10 groups were similar (Table 4). The average plasma SOD levels in the C2‐10, GER, and PGER groups were higher than those in the control group, but there were no significant differences among them. Compared with the control and C2‐10 groups, the kidney SOD levels were significantly higher in the GER and PGER groups (Table 4). The plasma MDA levels in the GER and PGER groups were significantly lower than those in the control group (Table 4). The kidney MDA levels in the C2‐10, GER, and PGER groups were significantly lower than those in the control group; however, there was no significant differences in the kidney MDA levels among the C2‐10, GER, and PGER groups. The plasma T‐AOC levels in the PGER group were significantly higher than those in the control group; however, the kidney T‐AOC levels were similar among the four groups (Table 4). The results suggested that diets supplemented with brown rice, especially giant embryo brown rice and germinated giant embryo brown rice, can enhance plasma and kidney antioxidant ability.

**Table 4:** Plasma and kidney antioxidant parameter levels (*n* = 10, $\overset{¯}{x}\pm \text{SEM}$)

Parameters	Groups			
	Control	C2‐10	GER	PGER
Plasma				
CAT (U/ml)	2.61 ± 0.34^a^	2.56 ± 0.26^a^	2.46 ± 0.22^a^	2.53 ± 0.33^a^
SOD (U/ml)	58.32 ± 2.12^a^	62.02 ± 3.17^a^	63.29 ± 2.65^a^	62.89 ± 1.37^a^
MDA (nmol/ml)	4.45 ± 0.47^aA^	3.59 ± 0.30^abAB^	2.79 ± 0.17^bcB^	2.51 ± 0.30^cB^
T‐AOC (U/ml)	1.33 ± 0.10^aA^	2.06 ± 0.23^abA^	1.96 ± 0.25^abA^	2.39 ± 0.36^bA^
Kidney				
CAT (U/ml)	15.68 ± 1.00^aA^	18.36 ± 1.46^abA^	21.74 ± 1.59^bAB^	26.80 ± 2.50^cB^
SOD (U/ml)	216.97 ± 20.38^aA^	241.91 ± 14.18^abAB^	351.20 ± 21.78^cBC^	405.91 ± 36.11^cC^
MDA (nmol/ml)	0.32 ± 0.02^A^	0.23 ± 0.01^B^	0.23 ± 0.02^B^	0.23 ± 0.01^B^
T‐AOC (U/ml)	0.56 ± 0.04^a^	0.52 ± 0.06^a^	0.64 ± 0.07^a^	0.63 ± 0.06^a^

Abbreviations: Control, control diet; C2‐10, “Chao2‐10” brown rice diet; GER, “Shangshida NO.5” giant embryo brown rice diet; PGER, pre‐germinated “Shangshida NO.5” giant embryo brown rice diet.

^a,b,c^ Mean values within a row with different superscript letters were significantly different (*p* < .05).

^A,B,C^ Mean values within a row with different superscript letters were significantly different (*p* < .01).

## 4 DISCUSSION

γ‐Aminobutyric acid is a nonprotein amino acid that acts as a major inhibitory neurotransmitter in the central nervous system. Based on the fact that GABA accumulates in “Shangshida NO.5” giant embryo brown rice (GER) and germinated “Shangshida NO.5” giant embryo brown rice (PGER; Zhao et al., 2017) and that foods and supplements containing GABA have obvious biological activities, including antihypertensive action (Matsubara, 2002; Tsuchida, 2003), it is plausible that diets containing high amounts of GER and PGER could have antihypertensive effects.

To test this hypothesis, in this study, the antihypertensive effects of diets supplemented with different brown rice derivatives were investigated, and the chronic administration of experimental diets containing C2‐10, GER, and PGER significantly decreased SBP in SHR (Figure 1). The GER and PGER groups showed significantly inhibited blood pressure elevation compared with the C2‐10 group. This result is consistent with higher GABA accumulation in “Shangshida NO. 5” giant embryo brown rice and germinated “Shangshida NO.5” giant embryo brown rice compared with “Chao2‐10” brown rice (Zhao et al., 2017). The antihypertensive effects of various foods rich in GABA have been reported. Mineka et al. found a decrease in SBP in SHR fed GABA‐enriched tomatoes (GABA: 178 mg/100 mg); however, this diet had no effect on DBP (Yoshimura et al., 2009). Nakamura (2000) found that a diet including GABA‐enriched chlorella (GABA: 33 mg/100 g) decreases SBP by 20 mmHg in SHR, but only tended to reduce DBP. In this study, considerable elevation‐inhibitory effects the on the DBP in SHR were observed in the GER and PGER groups (Figure 2). At the end of the experimental period, the DBP values in the C2‐10, GER, and PGER groups were 9.8, 21.1, and 29 mmHg less than that in the control group, respectively (Figure 2). Thus, C2‐10, GER, and PGER reduced both SBP and DBP, which is different from what was reported previously (Nakamura, 2000; Yoshimura et al., 2009). The above analysis suggested that C2‐10, GER, and PGER may contain other antihypertensive components. In addition, the DBP test results also supported that the GER and PGER diets had better hypotension effects than the C2‐10 diet (Figure 2). In this study, diets supplemented with “Chao2‐10” brown rice also significantly reduced SBP levels; however, in a previous report, no significant decrease in the SBP of SHR fed diets with conventional brown rice was observed (Kawakami et al., 2018). The discrepancy may be the result of the difference in the brown rice embryo volume between the two rice types. The “Chao2‐10” embryo volume is larger than that of conventional rice and is comparable to that of “M‐2‐565‐11–3‐B (*ge*),” a giant embryo rice developed in Korea (Ping et al., 2015).

The antihypertensive mechanism of GABA in SHR has not been fully explained, but some possible mechanisms have been postulated. In the peripheral vasculature, possible mechanisms include ganglionic blockade (Vemulapalli & Barletta, 1984), activation of GABAergic receptors (Vemulapalli & Barletta, 1984), direct action on the vasculature (Billingsley & Suria, 1982), and inhibition of transmitter release from sympathetic nerve terminals (Bowery et al., 1981; Manzini, Maggi, & Meli, 1985). Moreover, GABA could inhibit noradrenaline release from sympathetic nerve terminals within the mesenteric arterial bed of SHR (Hayakawa, Kimura, & Kamata, 2002). In addition, GABA inhibits blood pressure increase in sham‐operated SHR but not in renal sympathetic denervated SHR, which suggests that the antihypertensive effect of GABA may involve reducing rennin release. The GABA‐induced long‐term hypotensive effect in SHR may involve the following events: (a) GABA acting on GABAB receptors on the sympathetic nerve endings in the kidney to inhibit the release of noradrenaline; (b) reduced renin release; and (c) reduced angiotensin II formation owing to the decreased concentration of renin in plasma (Hayakawa, Kimura, & Yamori, 2005). In this study, the C2‐10, GER, and PGER diets had hypotensive effects on SHR, although it was not clear whether this effect was dependent on GABA. However, it seems reasonable to conclude that GABA plays an important role in the hypotensive effects of C2‐10, GER, and PGER. Our test results showed that the levels of NE and rennin in the C2‐10, GER, and PGER groups were significantly lower than those in the control group (Table 2). The levels of Ang II and ALD in the PGER group were significantly lower than those in the control group (Table 2), but the levels in the control, C2‐10, and GER groups were similar. Given that the GABA content in PGER is far higher than that in C2‐10 and GER (Zhao et al., 2017), the above results suggested that the stronger antihypertensive effect of the PGER diet may be derived from the higher GABA content in the pre‐germinated giant embryo brown rice.

In addition to GABA, giant embryo brown rice also contains more vitamin E, amino acids, and other nutrients (Wang et al., 2013; Zhang et al., 2005, 2007). We also found that there was 22.14% more dietary fiber in GER than in C2‐10 (data not shown). Previous reports showed that vitamin E has an antihypertensive effect and that supplementation with vitamin E (1,000 mg/day), vitamin C (1,000 IU/day), or tempol (1 mmol/day) for 6 weeks inhibits the development of hypertension and improves endothelial function in SHR (Park, Touyz, Chen, & Schiffrin, 2000). In addition, previous studies have also shown that several amino acids, such as tyrosine (Sved, Fernstrom, & Wurtman, 1979) and phenylalanine (Mitchell, Dorrance, & Webb, 2004), have antihypertensive effects. Feeding with a diet supplemented in dietary fiber‐rich rice for 8 weeks can significantly reduce SBP and DBP levels in SHR (Lee et al., 2004). Therefore, other components of the giant embryo brown rice, such as vitamin E, amino acids, and dietary fiber, may also contribute to the decreased SBP and DBP levels, as observed in this chronic study in SHR.

The kidney is the main target organ for hypertensive damage (Raij, 1986). Therefore, it is worth evaluating whether diets supplemented with these three kinds of brown rice can protect against the development of kidney disease. Based on the observed decreases in BUN and SCR levels (Table 3), diets supplemented with the three kinds of brown rice have obvious antihypertensive effects in SHR, and these effects may be due to their amelioration of kidney function. The beneficial effects of feeding diets supplemented with the three kinds of brown rice were further confirmed by the results of the plasma lipid parameters, including TG and TC (Table 3).

Oxidative stress plays an important role in the initiation and progression of cardiovascular diseases, including hypertension, type II diabetes, hypercholesterolemia, atherosclerosis, and heart failure (Hamilton et al., 2004). Reactive oxygen species (ROS) are prevalent in many tissues and organs. Excessive production of ROS is implicated in the development of hypertension and coronary heart disease at different stages, including vascular endothelial cell damage, foam cell formation, vascular smooth muscle cell proliferation, gene expression of blood pressure‐related factors, impaired vasomotor reactivity, and plaque instability (Giugliano, 2000; Kunsch & Medford, 1999). Previous research has shown that severe oxidative stress during hypertension is due to an imbalance of oxidant and antioxidant status, which also causes a reduction in antioxidant enzymes and increase in oxidation products (Koska et al., 1999; Newaz & Nawal, 1999). T‐AOC is an important indicator of oxidative stress injury in organisms, and it reflects the interlinkage and protective effect of antioxidants in vivo*.* SOD plays a key role in scavenging free radicals in vivo, and CAT works closely with SOD to prevent free radical damage to the body (Kumar & Das, 2000). In this study, the plasma T‐AOC levels in the PGER group SHR were significantly higher than those in the control group (Table 4). The kidney SOD levels and CAT activities in the GER and PGER groups were also significantly higher than those in the control group. We found that plasma MDA, a marker of lipid peroxidation induced by ROS, was significantly reduced in the GER and PGER groups compared with the control group (Table 4). However, the above parameters in the control group and the C2‐10 group are similar (Table 4). Moreover, the kidney SOD levels in the GER group were higher than those in the C2‐10 group (Table 4). A previous study showed that α‐tocopherol (a hydrolysate of vitamin E) may prevent the age‐related increases in blood pressure in SHR by elevating the total antioxidant status and SOD activity, which reduces lipid peroxidation (Newaz & Nawal, 1999). A previous study also found that “Shangshida NO.5” giant embryo brown rice is higher in vitamin E than “Chao2‐10” brown rice (Wang et al., 2013). Therefore, the vitamin E in giant embryo brown rice may contribute to improving the antioxidant capacity, as observed in this chronic study in SHR.

Altogether, our results showed that the administration of C2‐10, GER, and PGER significantly reduced SBP and DBP levels and ameliorated kidney function and antioxidant capacity. Given that brown rice and germinated brown rice can be eaten as part of a natural diet, GABA‐enriched “Shangshida NO.5” rice could be a good candidate for the prevention of hypertension.

## CONFLICT OF INTEREST

The authors declare that they do not have any conflict of interest.

## ETHICAL APPROVAL

“This study was approved by the Institutional Review Board of Shanghai Jiaotong University.”

## INFORMED CONSENT

Written informed consent was obtained from all study participants.
