# Supplementary material for: Antihypertensive effect of giant embryo brown rice and pre‐germinated giant embryo brown rice on spontaneously hypertensive rats
Source: Food Sci Nutr. 2019 Jul 30;7(9):2888–96. doi: 10.1002/fsn3.1137 (PMC6766545; doi:10.1002/fsn3.1137)
Supplement: Supplementary file 1 [file FSN3-7-2888-s001.docx]

Supplement Table 1 Different groups of rats weekly weight measurements in feeding period（n=10，$\bar{x}\pm\mathrm{SEM}$）

| (Weeks) | groups（g） | | | |
| --- | --- | --- | --- | --- |
|  | Control | C2-10 | GER | PGER |
| 0 | 228.6±6.8^aA^ | 222.5±1.0 ^aA^ | 229.0±4.1 ^aA^ | 229.6±7.1 ^aA^ |
| 1 | 260.2±5.1 ^aA^ | 256.4±1.3 ^aA^ | 258.0±3.9 ^aA^ | 258.3±6.0 ^aA^ |
| 2 | 279.8±5.8 ^aA^ | 275.7±1.6 ^aA^ | 279.4±4.2 ^aA^ | 275.6±5.9 ^aA^ |
| 3 | 299.0±5.4 ^aA^ | 296.0±1.7 ^aA^ | 295.0±4.1 ^aA^ | 293.7±6.3 ^aA^ |
| 4 | 312.5±5.1 ^aA^ | 306.2±3.1 ^aA^ | 304.5±4.7 ^aA^ | 303.6±6.3 ^aA^ |
| 5 | 325.8±5.9 ^aA^ | 323.7±2.0 ^aA^ | 312.3±4.7 ^aA^ | 317.3±5.2 ^aA^ |
| 6 | 336.5±6.4 ^aA^ | 328.3±1.8 ^aA^ | 323.0±4.7 ^aA^ | 331.5±5.8 ^aA^ |
| 7 | 345.1±6.9 ^aA^ | 341.0±1.9 ^aA^ | 332.4±4.1 ^aA^ | 339.3±5.1 ^aA^ |
| 8 | 351.0±7.2 ^aA^ | 350.7±1.6 ^aA^ | 339.2±2.4 ^aA^ | 343.6±4.6 ^aA^ |

Control, control diet; C2-10, “Chao2-10” brown rice diet; GER, “Shangshida NO.5” giant embryo brown rice diet; PGER, “Shangshida NO.5” germinated brown rice. ^a,b,c^ Mean values within a row with unlike superscript letters were significantly different (P＜0·05). ^A,B,C^ Mean values within a row with unlike superscript letters were significantly different (P＜0·01).

Supplement Table 2 Four groups of SHR weekly heart rate measurements in feeding period

（n=10，$\bar{x}\pm\mathrm{SEM}$）

| Weeks | groups（bpm） | | | |
| --- | --- | --- | --- | --- |
|  | Control | C2-10 | GER | PGER |
| 0 | 369.3±9.7^aA^ | 376.6±11.9 ^aA^ | 349.4±7.5 ^aA^ | 363.1±7.4 ^aA^ |
| 1 | 398.8±10.5 ^aA^ | 387.6±12.4 ^aA^ | 403.9±12.8 ^aA^ | 360.2±6.1 ^aA^ |
| 2 | 396.7±14.6 ^aA^ | 409.1±8.8 ^aA^ | 423.5±4.8 ^aA^ | 379.4±10.7 ^aA^ |
| 3 | 400.4±10.4 ^aA^ | 407.4±12.2 ^aA^ | 438.3±14.9 ^aA^ | 383.4±8.3 ^aA^ |
| 4 | 418.6±14.1 ^aA^ | 411.0±13.1 ^aA^ | 413.3±10.4 ^aA^ | 361.9±10.8 ^aA^ |
| 5 | 398.0±9.7 ^aA^ | 396.8±8.8 ^aA^ | 425.5±17.9 ^aA^ | 383.7±15.2 ^aA^ |
| 6 | 398.5±16.5 ^aA^ | 392.8±10.1 ^aA^ | 421.7±10.4 ^aA^ | 391.2±10.1 ^aA^ |
| 7 | 384.3±8.5 ^aA^ | 398.3±11.8 ^aA^ | 414.6±12.9 ^aA^ | 371.6±14.1 ^aA^ |
| 8 | 393.8±11.4 ^aA^ | 378.5±9.4 ^aA^ | 396.0±12.2 ^aA^ | 390.1±16.7 ^aA^ |

Control, control diet; C2-10, “Chao2-10” brown rice diet; GER, “Shangshida NO.5” giant embryo brown rice diet; PGER, “Shangshida NO.5” germinated brown rice. ^a,b,c^ Mean values within a row with unlike superscript letters were significantly different (P＜0·05). ^A,B,C^ Mean values within a row with unlike superscript letters were significantly different (P＜0·01).
